# Incidence, Risk Factors and Outcomes of Junctional Ectopic Tachycardia After Tetralogy of Fallot Repair in Pediatric Patients

**DOI:** 10.3390/children12111572

**Published:** 2025-11-19

**Authors:** Fatih Durak, Gokcen Ozcifci, Emine Pinar Kulluoglu, Ayse Berna Anil, Onur Isik, Muhammet Akyuz, Baris Guven

**Affiliations:** 1 Department of Pediatric Intensive Care Unit, Izmir Katip Celebi University Medical Faculty, Izmir 35030, Turkey; 2 Department of Pediatric Intensive Care Unit, Izmir City Hospital, Izmir 35030, Turkey; 3 Private Practice, 35540 Izmir, Turkey; 4 Izmir City Hospital, Department of Pediatric Heart Surgery, University of Health Sciences, Izmir 35030, Turkey; 5 Izmir City Hospital, Department of Pediatric Cardiology, University of Health Sciences, Izmir 35030, Turkey

**Keywords:** congenital heart disease, arrhythmia, junctional ectopic tachycardia, tetralogy of Fallot, pediatric intensive care

## Abstract

**Highlights:**

**What are the main findings?**

- Junctional ectopic tachycardia (JET) occurred in 16.7% of children after tetralogy of Fallot repair.
- Right ventricular outflow tract muscle resection, low ionized calcium, and higher vasoactive-inotropic scores were identified as independent risk factors for JET.

**What is the implication of the main finding?**

- Early identification of at-risk patients and optimization of perioperative electrolyte balance and hemodynamic management may reduce JET incidence and complications.
- Understanding modifiable surgical and metabolic risk factors can improve postoperative outcomes and survival in pediatric congenital heart surgery.

**Abstract:**

Background: Postoperative junctional ectopic tachycardia (JET) is a potentially life-threatening arrhythmia that may occur after congenital heart surgery, especially following tetralogy of Fallot (TOF) repair. It can cause hemodynamic instability due to atrioventricular dissociation. This study aimed to evaluate the incidence, risk factors, and outcomes of JET after TOF repair, with particular focus on management strategies and the impact of JET duration on recovery. Methods: This retrospective study included 114 pediatric patients who underwent TOF repair between 2015 and 2023. The study was approved by the institutional ethics committee (File No: 2023/09-31, Date: 10 October 2023). Postoperative JET was diagnosed based on standard electrocardiographic criteria. Perioperative variables, surgical techniques, and postoperative outcomes were analyzed. Results: JET occurred in 19 patients (16.7%). Compared with patients without JET, those with JET had higher complication rates (73.7% vs. 42.1%, *p* = 0.02), prolonged inotropic support, and increased mortality (15.8% vs. 2.1%, *p* = 0.024). Ionized calcium (*p* < 0.001) and pH levels (*p* < 0.037) were significantly lower in JET patients. Right ventricular outflow tract muscle resection was strongly associated with JET occurrence (*p* = 0.003). Although cardiopulmonary bypass and aortic cross-clamp times did not predict JET, both correlated with JET duration (*p* < 0.05). Conclusions: Postoperative JET remains a major concern following TOF repair, leading to adverse outcomes and longer recovery. Optimizing perioperative management may help reduce JET-related complications, though further multicenter prospective studies are needed to confirm these findings.

## 1. Introduction

Postoperative junctional ectopic tachycardia (JET), a potentially life-threatening arrhythmia, may occur following congenital cardiac surgery, particularly in pediatric patients undergoing repair for tetralogy of Fallot (TOF) [1]. JET, also referred to as His bundle tachycardia or junctional automatic tachycardia, is a non-reentrant tachyarrhythmia originating from the atrioventricular junction. It typically presents with a narrow QRS complex and may be regular or irregular, often showing retrograde atrial conduction or atrioventricular dissociation [2]. Its occurrence within 72 h after surgery is common, with manifestations of narrow QRS complexes, though bundle branch block presence, typical after TOF correction, may result in widened QRS complexes [1,3,4]. In rare instances, JET has been documented up to 50 days post-surgery [5].

The incidence of JET following congenital heart surgery varies widely, ranging from 1–38% [1,2,5,6,7,8,9,10,11]. Notably, its occurrence is higher when interventions are near the atrioventricular node and bundle of Hiss, as in TOF, and complete atrioventricular canal repair [1,12].

Despite being generally self-limiting, JET can lead to severe hemodynamic deterioration due to the loss of atrioventricular (AV) synchrony and compromised ventricular filling post-surgery [13]. Management strategies involve measures such as analgesia, sedation, cooling for normothermia, minimizing inotropic support, addressing electrolyte imbalances, external pacing for AV compliance, and heart rate control with medications like amiodarone, dexmedetomidine, and/or beta-blockers [2,4,5,14,15,16,17,18].

This study aims to share our experience with postoperative JET after TOF repair, identify associated factors, and analyze patient outcomes, focusing on the impact of JET on the postoperative course. Moreover, recent studies have raised questions about the potential association between the time taken to achieve rate control and the length of hospital stay [15,19]. By examining patients with TOF, we aim to address this question, among others, and provide further insight into the etiology of JET.

## 2. Materials and Methods

### 2.1. Study Population

We conducted a retrospective analysis of patients who underwent surgical repair for tetralogy of Fallot between 2015 and 2023. Patients with significant postoperative arrhythmias other than junctional ectopic tachycardia—including sustained supraventricular tachycardia, atrial flutter, atrial fibrillation, ventricular tachycardia, ventricular fibrillation, or complete atrioventricular block requiring permanent pacing—were excluded. Additionally, patients with incomplete postoperative data or missing diagnostic criteria for junctional ectopic tachycardia in their medical records were excluded.

A total of 19 patients were excluded: one due to junctional bradycardia, one due to ventricular tachycardia, seven with complete atrioventricular block, one with supraventricular tachycardia not fulfilling junctional ectopic tachycardia criteria, and ten due to incomplete postoperative data. Finally, 114 patients met the inclusion criteria and were included in the study.

### 2.2. Operative Technique

Surgery during these eight years was performed by the same surgeons with similar techniques and under standard cardiopulmonary bypass (CPB) conditions with cold crystalloid cardioplegia. Bicaval cannulation was performed, and cardioplegic arrest was achieved by antegrade cold crystalloid cardioplegia. The median temperature of the perfusate was 32 °C. In all cases, ventricular septal defect closure was performed by either a transatrial or transventricular approach using a patch of autologous pericardium or polytetrafluoroethylene (ePTFE). In most cases, the atrial septal defect (ASD) was completely closed, and in 27 cases, there was a small atrial communication (3–4 mm) left to avoid right ventricular dilatation. A commissurotomy or valvulotomy based on Z score with Hegar dilation was performed in patients with mild or mid-pulmonary stenosis. Infundibular muscle resection was performed in all patients. The right ventricular outflow tract (RVOT) was reconstructed with an autologous pericardium or a patch of ePTFE. Patients who required trans-annular patch (TAP) were mainly operated upon during the early study period, and our current strategy is to avoid TAP and preserve the pulmonary valve when possible.

### 2.3. Data Collection

We comprehensively examined preoperative, perioperative, and postoperative data, incorporating information from echocardiography reports, perfusion reports, and clinical, inpatient, and operative notes.

Patients’ medical charts were retrospectively reviewed to collect preoperative variables, including patients’ age at the time of surgery, gender, weight (kg), preoperative B-blockers administration, associated cardiac anomalies, and previous palliative surgical procedures as modified Blalock-Tausig Shunt. Preoperative 12-lead electrocardiogram was reviewed in all patients to identify the preoperative rhythm and PR interval, and QRS duration was calculated. Preoperative heart rate was reported from the anesthesia records after sedating the patients.

Intraoperative variables include the time (minutes) for CPB, the time (minutes) for aortic cross-clamp (ACC), and the type of surgical intervention.

Postoperative variables included in-hospital death, duration of mechanical ventilation, non-invasive ventilation, pediatric ICU stay, hospital stay, and mortality and organ dysfunction scores. The Pediatric Index of Mortality-2 (PIM-2) and Pediatric Risk of Mortality-3 (PRISM-3) scores were used to assess predicted mortality risk, while the Pediatric Logistic Organ Dysfunction (PELOD) score was used to evaluate the degree of organ dysfunction. Requirements for extracorporeal life support (ECLS) and continuous renal replacement therapy (CRRT), as well as the use of inotropic support, were recorded. The inotropic score (IS) and vasoactive-inotropic score (VIS) were calculated during the first 24 h to quantify the degree of pharmacologic cardiovascular support, where higher scores indicate greater inotropic and vasoactive agent requirements.

The postoperative complications encompass acute kidney injury, low cardiac output syndrome (LCOS), acute liver dysfunction (ALD), bypass-related systemic inflammatory response syndrome (SIRS), pleural effusion requiring chest tube placement, atelectasis, and hemorrhagic complications. Acute kidney injury (AKI) is defined by the pRIFLE (pediatric risk, injury, failure, loss, end-stage renal disease) criteria [20]. LCOS is defined as the use of ≥3 inotropes and is associated with the following: tachycardia, oliguria, decreased skin perfusion, metabolic acidosis, or vasopressin requirement for hypotension and/or shock in the postoperative period [21]. ALD was defined as those who met the Pediatric Organ Dysfunction Information Update Mandate (PODIUM) criteria, including aspartate aminotransferase > 100 IU/L or alanine aminotransferase > 100 IU/L or glutamyl transpeptidase > 100 IU/L or total bilirubin > 5 mg/dL or direct or conjugated bilirubin > 2 mg/dL [22]. SIRS was determined according to international pediatric sepsis consensus criteria, that is, the presence of at least two of the following four criteria with either abnormal temperature or leukocyte count as an obligate criterion: core temperature of >38.5 °C or <36 °C, tachycardia (irrespective of inotropic support) defined as mean heart rate > 2 standard deviations (SD) beyond normal for age, mean respiratory rate > 2 SD above normal for age, elevated or reduced age-specific leukocyte count or >10% immature neutrophils. Bypass-related SIRS is defined as SIRS that occurs within the first 48 h after bypass, where no other etiological causes are detected [23,24].

Preoperative and postoperative hemoglobin values, calcium (corrected), sodium, potassium, magnesium, ionized calcium, pH, and lactate (within the first 1 h) levels of the patients were collected.

The primary endpoint was the incidence of postoperative JET. Secondary endpoints included identification of perioperative risk factors, JET duration, postoperative complications, and mortality.

### 2.4. Postoperative JET Approaching

We included all patients who had JET (19 patients), regardless of its duration, and determined its onset as either intraoperative or postoperative in the PICU. The diagnosis of JET was confirmed by a pediatric cardiologist in the PICU using a 12-lead electrocardiogram (ECG). Diagnostic criteria included (i) heart rate greater than +2 standard deviations for age; (ii) absent P wave in lead II; (iii) narrow QRS complexes; and (iv) ventricular rate faster than atrial rate with atrioventricular dissociation. When P-wave identification was uncertain, atrial/ventricular pacing wires and 12-lead ECG were reviewed by a pediatric cardiologist.

JET management followed a stepwise protocol. Conventional methods were defined as non-pharmacological measures, including discontinuation of unnecessary inotropes, administration of intravenous fluid boluses for hypovolemia (central venous pressure < 5 mmHg or clinical signs of low preload), surface cooling to 36–36.5 °C, and sedation. Electrolyte imbalances were corrected aggressively to maintain serum potassium > 4.0 mmol/L, ionized calcium > 1.1 mmol/L, and serum magnesium > 1.6 mg/dL. If JET persisted despite these measures, amiodarone was administered as a bolus of 5 mg/kg over one hour, followed by a continuous infusion of 5–15 μg/kg/min until sinus rhythm was restored or heart rate slowed to a hemodynamically acceptable range. Beta-blockers, particularly esmolol, were used in selected cases but not as first-line therapy. For postoperative pacing, a ventricular pacing wire was routinely inserted unless heart block occurred immediately after separation from CPB, in which case both atrial and ventricular pacing wires were placed.

### 2.5. Statistical Analyses

Statistical analyses were performed using the Statistical Package for the Social Sciences version 24.0 software (Armonk, NY, USA: IBM Corp). Normal distribution was assessed using visual (histogram and probability graphs) and analytical methods (Kolmogorov–Smirnov and Shapiro–Wilk test). Descriptive analysis employed frequency tables for categorical variables, with means and standard deviations for normally distributed variables and medians and ranges for those with a non-normal distribution. For categorical variables, Chi^2^ was used or the Fisher Exact test if the frequency is less than 5. Differences in continuous variables were compared with the nonparametric Mann–Whitney U test. To examine the relationship between continuous variables, the Spearman rank correlation was used. Variables showing a significant association in the univariate analysis were considered for inclusion in the multivariate model. Multivariable analysis was performed using linear logistic regression to identify independent predictors and outcomes of JET. During this analysis, preoperative and operative risk factors were analyzed in one model, postoperative factors in another model, and outcomes in a separate model. A *p*-value of less than 0.05 was considered statistically significant.

## 3. Results

A total of 114 patients underwent TOF repair during the study period. Demographic characteristics, preoperative, and operative data are presented in Table 1. The median age at surgery was 10 months (IQR 6–17.25), and the median body weight was 8 kg (IQR 6.8–10). Sixty-four patients (56.1%) were male. The most common associated cardiac defects were atrial septal defect (*n* = 14, 12.3%) and patent ductus arteriosus (*n* = 4, 3.5%). Seventeen patients (14.9%) had pre-existing conditions, with Down syndrome (*n* = 6, 5.3%) being the most frequent genetic anomaly. Ten patients (8.8%) had craniofacial anomalies.

In the preoperative period, 67 (58.8%) patients were on beta-blockers. Preoperative echocardiography showed a mean pulmonary annulus diameter (PAD) of 8.1 ± 2.1 mm and an RVOT pressure gradient of 80.1 ± 17.3 mmHg. Thirteen patients (11.4%) had previously undergone modified Blalock–Taussig–Thomas (mBTT) shunt placement. The mean CPB time was 103 ± 20 min, and the mean ACC time was 80 ± 17 min. Surgical techniques included transannular patching in 95 (83.3%) patients, RVOT muscle resection in 93 (81.6%), pulmonary valvotomy in 45 (39.5%), conduit repair between the right ventricle and pulmonary artery in 7 (6.1%), and right ventriculotomy in 51 (44.7%).

JET occurred in 19 (16.7%) patients and was more frequent in those who underwent RVOT muscle resection (20.4% vs. 0%, *p* = 0.022). No significant association was found between JET and other surgical approaches, including transannular patching, pulmonary valvotomy, conduit repair, or right ventriculotomy. Dexmedetomidine was administered postoperatively in 90 (78.9%) patients, with 2 (1.8%) receiving it intraoperatively and 22 (19.3%) not receiving it at all. There was no statistically significant association between the presence of genetic syndromes, including Down syndrome, and the occurrence of JET (*p* = 0.990). Similarly, neither associated cardiac (*p* = 0.613) nor non-cardiac anomalies (*p* = 0.830) were related to JET incidence.

Table 2 summarizes the complications and outcomes of the patients. The overall complication rate was significantly higher in the JET group (73.7% vs. 42.1%, *p* = 0.022). Patients with JET had a significantly higher incidence of ALD (36.8% vs. 7.4%, *p* = 0.002), LCOS (31.6% vs. 7.4%, *p* = 0.007), and bypass-related SIRS (21.1% vs. 4.2%, *p* = 0.02), while no significant differences were observed in other complications. Although AKI occurrence was similar between groups, patients with JET who developed AKI had a significantly higher need for CRRT (42.9% vs. 10.5%, *p* = 0.024). Overall, 5 (4.4%) patients required CRRT, 6 (5.3%) required ECLS, and 5 (4.4%) died. JET was associated with a significantly higher mortality rate (15.8% vs. 2.1%, *p* = 0.024). Causes of death included junctional bradycardia leading to sudden cardiac arrest (*n* = 1), LCOS despite JET resolution (*n* = 2), LCOS in a non-JET patient (*n* = 1), and Candida-related septic shock during prolonged PICU stay (*n* = 1).

Preoperative mBTT shunt placement (*p* = 0.121), beta-blocker use (*p* = 0.447), postoperative dexmedetomidine use (*p* = 0.399), and type or number of inotropic agents (*p* = 0.228) did not influence JET development.

Among the 19 patients diagnosed with JET, 10 (52.6%) developed JET intraoperatively, 7 (37.1%) developed JET within the first 24 h, 1 patient developed JET at 70 h, and 1 at 144 h. The average JET duration was 14.42 ± 8.29 h. All patients with JET were treated with conventional methods, magnesium sulfate, and dexmedetomidine, with 10 (52.6%) recovering successfully. The rhythm was restored in 7 (36.8%) with amiodarone and 2 (10.5%) with additional beta-blockers.

Patients with JET had significantly lower ionized calcium (1.15 ± 0.1 mmol/L vs. 1.05 ± 0.1 mmol/L, *p* < 0.001) and lower pH (7.33 ± 0.01 vs. 7.29 ± 0.18, *p* = 0.037), while no significant differences were observed in other laboratory values (Table 3). Patients with JET had higher inotropic scores (IS: 14 [IQR 10–20] vs. 10 [IQR 5–15], *p* = 0.016), higher vasoactive-inotropic scores (VIS: 15 [IQR 10–30] vs. 10 [IQR 5–15], *p* = 0.009), longer inotropic support duration (30 [IQR 24–48] hours vs. 14 [IQR 10–24] hours, *p* < 0.001), and longer mechanical ventilation (MV) duration (4 [IQR 3–32] hours vs. 3 [IQR 2–4] hours, *p* = 0.017). Following multivariate analysis, lower ionized calcium and lower pH remained independently associated with an increased risk of JET (Table 4). To address the potential for both reverse causality (temporal bias) and confounding by operative complexity (RVOT resection), a final, stringent sensitivity analysis was conducted. This model excluded immediate-onset JET cases (<1 h) and included RVOT muscle resection status as a mandatory adjustment variable (*n* = 104 cases). The results of this doubly adjusted model demonstrated high robustness: low ionized calcium maintained its highly significant protective effect, independent of surgical complexity (OR = 0.000; 95% CI: 0.000–0.001, *p* = 0.004). The protective effect of pH, however, lost statistical significance in this model (*p* = 0.117).

In the correlation analysis (Table 5), JET duration was significantly correlated with CPB time (r = 0.466, *p* = 0.044), ACC time (r = 0.479, *p* = 0.038), PRISM score (r = 0.461, *p* = 0.047), and PICU length of stay (r = 0.527, *p* = 0.02). A significant negative correlation was found between JET onset time and MV duration (r = −0.525, *p* = 0.021), suggesting that earlier-onset JET is associated with prolonged ventilation.

Given the relatively small number of JET cases (*n* = 19), effect sizes and 95% confidence intervals were not calculated. Instead, statistical significance was assessed using *p*-values, and findings should be interpreted with caution due to potential imprecision.

## 4. Discussion

Postoperative JET remains a significant concern following TOF repair due to its association with adverse outcomes, including hemodynamic instability and prolonged PICU stay. While its exact etiology remains unclear, JET is believed to result from a combination of congenital heart defects and the effects of surgical intervention. The proposed mechanism involves direct mechanical trauma or indirect stretch-related injury to the conduction system, particularly in procedures involving the right ventricular outflow tract [3,25].

In our study, JET occurred in 16.7% (19/114) of patients, aligning with previously reported incidence rates ranging from 1% to 38% in congenital heart surgery, especially in procedures involving structures near the AV node [1,5,7,8,9,10,11,14]. Differences in study design and patient selection likely explain the variability in reported incidence. While some studies have identified younger age and lower body weight as risk factors for JET, our findings did not support this association [5,9,10,11]. Previous studies have linked prolonged ACC and CPB times to an increased risk of JET. However, in our study, these factors were not identified as significant risk factors for JET development. This discrepancy may be attributed to the relatively shorter ACC and CPB durations in our cohort. Although no definitive cut-off values exist in the literature, studies suggest that CPB times exceeding 90–120 min are more frequently observed in patients who develop JET [5,8,9,10,11,25]. Notably, while ACC and CPB times did not predict JET occurrence in our study, we found a significant positive correlation between JET duration and both ACC and CPB times, a relationship that has not been emphasized in previous research. This suggests that while longer operative times may not directly trigger JET, they could contribute to its persistence.

Although genetic syndromes have been linked to increased complication rates following congenital heart surgery, specific data on their association with JET remain limited [26,27]. In our study, genetic syndromes did not significantly impact JET occurrence, but our data on this topic were limited. Further research is needed to clarify this potential relationship.

In patients with JET, IS and VIS were significantly higher, and the duration of inotropic agent use was longer, consistent with previous reports [10,11,28,29]. We interpret this strong statistical association as signifying that severe underlying postoperative stress and clinical intervention are strongly related to JET development. We acknowledge that the relationship here signifies a strong correlation with severity rather than a strict causal factor. However, preoperative beta-blocker use, postoperative dexmedetomidine use, and the type or number of inotropic agents did not influence JET occurrence. This contrasts with studies suggesting a protective effect of dexmedetomidine and beta-blockers in reducing JET incidence [15,18,30]. Another study reported findings similar to ours, indicating that inotropic agents did not affect JET development [31]. Conversely, one study identified a specific association between norepinephrine use and JET, highlighting potential discrepancies due to differences in patient selection, surgical techniques, and dosing protocols [32]. Additionally, consistent with the findings of Ismail et al., no association was found between PAD, RVOT pressure gradient, and either the presence or duration of JET [31].

Electrolyte imbalances have been frequently debated as potential contributors to JET. While some studies have suggested a role for serum magnesium as either a risk factor or a prophylactic treatment for JET [31,33,34], others have found no significant differences in magnesium and calcium levels between JET and non-JET groups [8]. Additionally, studies indicate that potassium does not significantly impact JET occurrence [32]. Our findings suggest that lower ionized calcium and pH levels were associated with JET development, while magnesium and potassium had no significant effect. A key limitation of our retrospective study is establishing definitive causality, given the strong association between early markers and operative complexity (RVOT resection). To address this, we conducted a rigorous sensitivity analysis controlling simultaneously for temporal bias (excluding immediate JET onset) and procedural confounding (RVOT adjustment). The persistence of a highly significant and protective effect for ionized calcium in this highly controlled model is a strong validation. This evidence supports the hypothesis that low early post-operative ionized calcium is an independent, predisposing risk factor for delayed JET, rather than a mere consequence of the arrhythmia or surgical complexity. We acknowledge that the effect of pH was confounded by the inclusion of RVOT resection and became non-significant. However, the stable significance of ionized calcium—a closely related factor—maintains the biological plausibility that early biochemical status is critical. Additionally, due to the high risk of collinearity and circular definition associated with VIS/IS, these variables were excluded from the final multivariable analysis. Finally, our analysis showed no association between JET and levels of preoperative and postoperative hemoglobin, lactate, or sodium, factors that have not been extensively evaluated in the literature.

Surgical trauma remains a major consideration in JET development. Damage to the conduction system can occur due to RVOT muscle resection, muscle bundle excision, or relief of RVOT obstruction, procedures often required in TOF repair [35]. In our study, JET was significantly more frequent in patients who underwent RVOT muscle resection, supporting prior findings that surgical trauma contributes to JET pathogenesis. However, transannular patching, pulmonary valvotomy, conduit repair, and right ventriculotomy were not significantly associated with JET development. Further research is needed to evaluate the role of different surgical techniques in JET occurrence.

Most cases of JET reported in the literature resolve with conventional methods and are considered self-limiting [3,9]. One study reported that 40% of cases resolved with dexmedetomidine and conventional methods, while 52% required amiodarone [11]. Another study found that 57% of cases resolved with conventional methods and magnesium, with the remaining 43% responding to amiodarone, leading to complete resolution [25]. In our study, a similar rate of improvement (52.6%) was observed with conventional methods, magnesium sulfate, and dexmedetomidine use, while an additional 36.8% of cases resolved with amiodarone, and 10.5% with beta-blocker therapy. While we did not find a need for additional pharmacologic interventions, other studies have documented the effectiveness of flecainide, procainamide, propafenone, sotalol, nifekalant hydrochloride, and landiolol hydrochloride in JET treatment [16,17,36,37,38].

The time required to achieve JET rate control varies significantly across studies. Hoffman et al. reported a mean time of 1.7 ± 1.7 days, while some single-center studies documented faster control times [5]. A retrospective study of 15 patients achieved a median rate control time of 4.5 (1.0–19.5) hours, while a prospective study of 71 patients reported a median of 3.0 (1.0–69.0) hours. Another study found a mean rate control time of 1 h [39,40,41]. Apart from all these studies, the median duration of JET was around 30.5–33.8 h in various publications [25,31]. In our cohort, the mean JET duration was 14.42 ± 8.29 h, shorter than in some studies but longer than in others. These variations likely stem from differences in JET management strategies. While some studies focus on heart rate control, others assess the time required to restore sinus rhythm, contributing to discrepancies in reported outcomes. Additionally, variations in treatment protocols may further explain these differences.

In most studies, JET is observed within the first 24 h postoperatively, and our findings were consistent with this trend [2,9]. In our cohort, 52.6% of JET cases developed intraoperatively, 37.1% within 24 h, and 10.5% had a delayed onset at 70 and 144 h postoperatively. The presence of such late-onset cases highlights that, although rare, JET can occur much later in the postoperative period.

Patients with JET had significantly higher complication rates and were more likely to develop multiorgan failure secondary to LCOS [9,11,32,35]. Prior studies have examined the relationship between amiodarone use and ALD, as well as dexmedetomidine and AKI, but detailed statistical analyses on individual complications remain limited [42,43]. Our analysis revealed that the overall complication rate was significantly higher in patients with JET. ALD, LCOS, and bypass-related SIRS were significantly more common, while no significant differences were observed in other complications. Although AKI incidence was similar between groups, patients with JET who developed AKI had a higher need for CRRT.

The role of body temperature in JET remains controversial. Some studies have reported no association, while others have suggested that elevated body temperature may contribute to JET onset [31]. In our study, similar to the findings of Mildh et al., body temperature was significantly higher in the JET group [32]. However, whether this elevation triggers JET onset or occurs as a consequence of JET remains unclear. A limitation of our study was the lack of continuous 24 h central body temperature measurements, which may have provided more precise data.

Mortality risk stratification tools have not been extensively studied in JET patients. In our study, the PRISM score was positively correlated with JET duration, while the PELOD and PIM scores were not significantly associated with JET presence or duration. The relationship between mortality risk scores and JET has not been previously evaluated, and further studies may help refine prognostic assessment models.

Although most studies have shown that JET increases mortality [3,9,11,25,35], a few have found no significant impact [32]. Similarly, while prolonged ICU stay, hospital stay, and MV duration are commonly associated with JET [5,9,11,25,32,35], some studies have reported contradictory findings [31]. In our study, the duration of MV support was longer, and mortality was higher in patients with JET compared to the non-JET group. Although JET presence itself did not correlate with longer ICU or hospital stays, a positive correlation was observed between JET duration and PICU length of stay. Additionally, a negative correlation was found between MV duration and JET onset time, suggesting that earlier-onset JET is linked to prolonged ventilation. Correlations involving JET duration were exploratory and should be considered hypothesis-generating due to the small sample size.

## 5. Limitations

This study has several limitations. First, as a single-center retrospective analysis using a complete-case approach (excluding cases with missing data) for multivariable modeling, it may introduce selection bias and limit the generalizability of the findings. Second, the relatively small number of patients with JET (*n* = 19) restricts statistical precision and contributes to wide confidence intervals; therefore, these observations should be interpreted as hypothesis-generating rather than confirmatory. Third, VIS and IS were excluded from the final multivariable model due to the high risk of collinearity and circular definitions with LCOS, reducing the potential for model bias. Fourth, while pH and ionized calcium values were collected early in the postoperative period, they may reflect physiological consequences rather than causal factors. Finally, surgical strategies and perioperative management evolved throughout the study period, which could have influenced outcomes. However, a sensitivity analysis controlling for both temporal bias (excluding JET onset ≤ 1 h) and procedural confounding (RVOT adjustment) confirmed that the association between low ionized calcium and JET remained robust and independent.

## 6. Conclusions

JET is one of the most common hemodynamically significant arrhythmias following congenital heart surgery. While many of the predictors we studied are interrelated, the highest risk factors for JET after TOF correction include high VIS, high IS, ionized calcium, low pH, and RVOT muscle resection. JET following TOF repair is linked to higher complication rates, longer mechanical ventilation, and increased mortality. While our findings support optimizing perioperative management, further multicenter studies are necessary to develop targeted prevention and treatment strategies.

## Figures and Tables

**Table 1:** Demographic, preoperative, and operative data in JET and non-JET patients.

Variable	JET Group	Non-JET	All Patient	*p*
Male, n (%)	13 (68.4%)	51 (53.7%)	64 (56.1%)	0.31
Age (months), median (IQR 25–75p)	9 (6–18)	11 (7–14)	10 (6–17.25)	0.49
Weight (kg)	7.4 (6–9)	8 (7–10)	8 (6.8–10)	0.17
Concomitant cardiac defects, n (%)	3 (15.8%)	18 (18.9%)	21 (18.4%)	0.613
Non-cardiac anomalies, n (%)	1 (5.3%)	14 (14.8%)	15 (13.2%)	0.83
Pre-existing diseases, n (%)	3 (15.8%)	14 (14.8%)	17 (14.9%)	0.99
*Down syndrome*	1 (5.3%)	5 (5.3%)	6 (5.3%)	
*Other genetic syndromes*	2 (10.5%)	9 (9.5%)	11 (9.6%)	
Preoperative beta-blocker user, n (%)	13 (68.4%)	54 (56.8%)	67 (58.8%)	0.447
Previous mBTT shunt, n (%)	0 (0%)	13 (13.7%)	13 (11.4%)	0.121
PAD (mm), mean ± SD	7.9 ± 1.9	8.1 ± 2.1	8.1 ± 2.1	0.78
RVOT pressure gradient (mmHg), mean ± SD	76 ± 20	81.9 ± 16.6	80.1 ± 17.3	0.18
CPB time (minutes), mean ± SD	105 ± 20	103 ± 20	103 ± 20	0.73
ACC time (minutes), mean ± SD	81 ± 17	80 ± 18	80 ± 17	0.81
Transannular patching, n (%)	17 (89.5%)	78 (82.1%)	95 (83.3%)	0.68
**RVOT muscle resection, n (%)**	**19 (100%)**	74 (77.9%)	93 (81.6%)	**0.003**
Conduit between the right ventricle and pulmonary artery, n (%)	1 (5.3%)	6 (6.3%)	7 (6.1%)	0.86
Right ventriculotomy, n (%)	11 (57.9%)	40 (42.1%)	51 (44.7%)	0.22
Pulmonary valvotomy, n (%)	8 (42.1%)	37 (38.9%)	45 (39.5%)	0.80

Bold shows statistically significance.

**Table 2:** Postoperative and outcomes data in JET and non-JET patients.

Variable	JET Group	Non-JET	All Patient	*p*
**Complications, n (%)**	**14 (73.7%)**	40 (42.1%)	54 (47.4%)	**0.02**
AKI, n (%)	7 (36.8%)	19 (20%)	26 (22.8%)	0.13
**ALD, n (%)**	**7 (36.8%)**	7 (7.4%)	14 (12.3%)	**0.002**
* Pulmonary complications, n (%)	4 (21.1%)	24 (25.7%)	28 (24.6%)	0.59
**LCOS, n (%)**	**6 (31.6%)**	7 (7.4%)	13 (11.4%)	**0.007**
**Bypass related SIRS, n (%)**	**4 (21.1%)**	4 (4.2%)	8 (7%)	**0.02**
Hemorrhagic complications, n (%)	0 (0%)	2 (2.1%)	2 (1.8%)	0.39
Convulsion, n (%)	1 (5.3%)	1 (1.1%)	2 (1.8%)	0.27
Sepsis, n (%)	2 (10.5%)	9 (9.5%)	11 (9.6%)	0.89
**Fever, n (%)**	**6 (31.6%)**	7 (7.4%)	13 (11.4%)	**0.007**
**CRRT, n (%)**	**3 (15.8%)**	2 (2.1%)	5 (4.4%)	**0.024**
ECLS, n (%)	2 (10.5%)	4 (4.2%)	6 (5.3%)	0.3
PIM, median (IQR 25–75p)	4.4 (2.7–10.5)	4.1 (2–6.5)	4.1 (2.1–7)	0.09
PRISM, median (IQR 25–75p)	7 (3–10)	5 (2–11)	6 (2.8–10.3)	0.33
PELOD, median (IQR 25–75p)	11 (2–21)	2 (1–12)	2 (1–12)	0.175
**VIS, median (IQR 25–75p)**	**15 (10–30)**	10 (5–15)	10 (5–15.9)	**0.009**
**IS, median (IQR 25–75p)**	**14 (10–20)**	10 (5–15)	10 (5–15)	**0.016**
**Inotropic agent time (hours), median (IQR 25–75p)**	**30 (24–48)**	14 (10–24)	18 (10–30)	**<0.001**
Postoperative dexmedetomidine use, n (%)	16 (84.2%)	74 (77.9%)	90 (78.9%)	0.399
PICU stay (days), median (IQR 25–75p)	5 (3–10)	4 (3–6)	5 (3–6)	0.066
**MV time (hours), median (IQR 25–75p)**	**4 (3–32)**	3 (2–4)	3 (2–5)	**0.017**
**Mortality, n (%)**	**3 (15.8%)**	2 (2.1%)	5 (4.4%)	**0.024**

* pleural effusion requiring chest tube placement, atelectasis, and chylothorax. Bold means that *p* value < 0.05 and this is statistically significant.

**Table 3:** The laboratory values in JET and non-JET patients.

Variable (All Value Is Mean ± SD)	JET Group	Non-JET	All Patient	*p*
Preoperative hemoglobin (g/dL)	13.9 ± 1.6	13.5 ± 2.4	13.5 ± 2.3	0.460
Postoperative hemoglobin (g/dL)	11.2 ± 1.8	11.4 ± 1.4	11.4 ±1.5	0.541
Sodium (mmol/L)	144 ± 4.1	143.5 ± 3.1	143.6 ± 3.3	0.709
Potassium (mmol/L)	4 ± 0.5	3.9 ± 0.5	3.9 ± 0.5	0.117
Magnesium (mg/dL)	3.2 ± 0.6	3.1 ± 0.6	3.1 ± 0.6	0.782
Corrected calcium (mg/dL)	8.4 ± 0.6	8.4 ± 0.7	8.4 ± 0.6	0.793
**Ionized calcium (mmol/L)**	**1.04 ± 0.11**	1.15 ± 0.10	1.14 ± 0.11	**<0.001**
**pH**	**7.29 ± 0.08**	7.33 ± 0.08	7.33 ± 0.08	**0.037**
Lactate (mmol/L)	2.04 ± 0.6	1.85 ± 0.9	1.88 ± 0.9	0.386

Bold shows statistically significance.

**Table 4:** Factors affecting JET in the multivariable analysis.

Variable	* OR (Exp(B))	95% * CI	*p*	* VIF
**Ionized calcium (mmol/L)**	0.000	0.000–0.001	**<0.001**	1.5
**pH**	0.000	0.000–0.143	**0.016**	1.1
*Final Sensitivity Analysis (Adjusted for Temporal Bias and RVOT)*				
**Ionized calcium (mmol/L)**	0.000	0.000–0.001	**<0.001**	1.6
pH	0.000	0.000–14.883	0.117	1.1

* OR odds ratio, CI confidence interval, VIF Variance Inflation Factor. Bold shows statistically significance.

**Table 5:** Correlation analysis.

Variable	Onset Time of JET (Hours)	JET Duration (Hours)
Body weight (kg)	r: 0.097, *p*: 0.693	r: −0.249, *p*: 0.304
Age (months)	r: 0.193, *p*: 0.428	r: 0.067, *p*: 0.785
PAD (mm)	r: 0.088, *p*: 0.720	r: −0.293, *p*: 0.223
RVOT pressure gradient (mmHg)	r: 0.136, *p*: 0.579	r: 0.236, *p*: 0.330
**CPB time (minutes)**	r: 0.181, *p*: 0.457	**r: 0.466, *p*: 0.044**
**ACC time (minutes)**	r: 0.118, *p*: 0.629	**r: 0.479, *p*: 0.038**
PIM	r: −0.185, *p*: 0.448	r: 0.307, *p*: 0.202
**PRISM**	r: −0.270, *p*: 0.264	**r: 0.461, *p*: 0.047**
PELOD	r: −0.011, *p*: 0.964	r: 0.155, *p*: 0.525
IS	r: −0.175, *p*: 0.474	r: 0.399, *p*: 0.090
VIS	r: −0.224, *p*: 0.356	r: 0.430, *p*: 0.066
**PICU stay (days)**	r: −0.096, *p*: 0.695	**r: 0.527, *p*: 0.020**
**MV time (hours)**	**r: −0.525, *p*: 0.021**	r: 0.357, *p*: 0.134

Bold shows statistically significance.

## Data Availability

No new data were created or analyzed in this study. Data sharing is not applicable to this article.
